# Anxiety, Anger and Depression Amongst Low-Income Earners in Southwestern Uganda During the COVID-19 Total Lockdown

**DOI:** 10.3389/fpubh.2021.590458

**Published:** 2021-12-09

**Authors:** Victor Archibong, Ibe Michael Usman, Keneth Iceland Kasozi, Eric Osamudiamwen Aigbogun, Ifie Josiah, Ann Lemuel Monima, Robinson Ssebuufu, Gaudencia Chekwech, Swase Dominic Terkimbi, Okon Owoisinke, Ngala Elvis Mbiydzenyuy, Azeez Adeoye, Joshua Ojodale Aruwa, Adam Moyosore Afodun, Saidi Odoma, Fred Ssempijja, Emmanuel Tiyo Ayikobua, John Tabakwot Ayuba, Viola Nankya, Comfort Onongha, Sussan Henry, Kevin Matama, Helen Yusuf, Halima Nalugo, Ewan MacLeod, Susan Christina Welburn

**Affiliations:** ^1^Department of Anatomy, College of Medicine, King Ceasor University, Kampala, Uganda; ^2^Faculty of Biomedicals Sciences, Kampala International University Western Campus, Bushenyi, Uganda; ^3^Infection Medicine, Deanery of Biomedical Sciences, College of Medicine and Veterinary Medicine, The University of Edinburgh, Edinburgh, United Kingdom; ^4^Uganda Medical and Dental Practitioners' Council, Kampala, Uganda; ^5^Faculty of Clinical Medicine and Dentistry, Kampala International University Teaching Hospital, Bushenyi, Uganda; ^6^Faculty of Allied Medical Sciences, University of Calabar, Calabar, Nigeria; ^7^Department of Basic Medical Science, School of Medicine, Copperbelt University, Ndola, Zambia; ^8^Department of Anatomy and Cell Biology, Faculty of Health Sciences, Busitema University, Busitema, Uganda; ^9^School of Pharmacy, Kampala International University Western Campus, Kampala, Uganda; ^10^Department of Physiology, School of Health Sciences, Soroti University, Soroti, Uganda; ^11^School of Nursing, Kampala International University Teaching Hospital, Bushenyi, Uganda; ^12^Department of Human Anatomy, College of Medicine and Health Science, Ahmadu Bello University, Zaria, Nigeria; ^13^Department of Anatomy, School of Medicine, Mbarara University of Science and Technology, Mbarara, Uganda; ^14^Zhejiang University-University of Edinburgh Institute, Zhejiang University School of Medicine, International Campus, Zhejiang University, Haining, China

**Keywords:** COVID-19 response in Africa, socio-economic impacts of COVID-19, COVID-19 outcomes, psychosocial impacts of COVID-19, hunger and COVID-19, COVID-19 hits poor harder, women dangers in COVID-19

## Abstract

**Background:** Low-income earners are particularly vulnerable to mental health, consequence of the coronavirus disease 2019 (COVID-19) lockdown restrictions, due to a temporary or permanent loss of income and livelihood, coupled with government-enforced measures of social distancing. This study evaluates the mental health status among low-income earners in southwestern Uganda during the first total COVID-19 lockdown in Uganda.

**Methods:** A cross-sectional descriptive study was undertaken amongst earners whose income falls below the poverty threshold. Two hundred and fifty-three (*n* = 253) male and female low-income earners between the ages of 18 and 60 years of age were recruited to the study. Modified generalized anxiety disorder (GAD-7), Spielberger's State-Trait Anger Expression Inventory-2 (STAXI-2), and Beck Depression Inventory (BDI) tools as appropriate were used to assess anxiety, anger, and depression respectively among our respondents.

**Results:** Severe anxiety (68.8%) followed by moderate depression (60.5%) and moderate anger (56.9%) were the most common mental health challenges experienced by low-income earners in Bushenyi district. Awareness of mental healthcare increased with the age of respondents in both males and females. A linear relationship was observed with age and depression (*r* = 0.154, *P* = 0.014) while positive correlations were observed between anxiety and anger (*r* = 0.254, *P* < 0.001); anxiety and depression (*r* = 0.153, *P* = 0.015) and anger and depression (*r* = 0.153, *P* = 0.015).

**Conclusion:** The study shows the importance of mental health awareness in low resource settings during the current COVID-19 pandemic. Females were identified as persons at risk to mental depression, while anger was highest amongst young males.

## Introduction

COVID-19 emerged in Africa on February 14th, 2020 with the first case reported in Egypt. To date 54 countries on the African sub-continent have now reported cases of COVID-19 (1). Uganda reported its first confirmed case on 22 March 2020, from a 36-year-old male who had traveled from Dubai (2, 3). On 18th March 2020, the President of Uganda announced the first total national lockdown which included the international border closures; the closing of schools, private offices and banned public gatherings at places of worships/social events, initially for a period of 32 days (4). Efforts to prevent the spread of COVID-19, effectively closed off most sources of income for the majority of low-income earners, who were forced to stay at home. The resulting decrease in household income impacted on food security and hunger and boredom have impacted on mental health and well-being, complicating adjustment to existence under the COVID-19 lockdown (5). Social distancing measures proposed by health experts and adopted by government lead to social isolation, especially in low-income settings where resources are limited and social networks are cut off by a lack of disposable income. Incidence of domestic violence has increased under conditions of social lockdown, exacerbated by economic uncertainty and stress; reports of domestic violence have been reported as tripling in some countries following previously reported increased rates of child abuse, neglect, and exploitation during previous public health emergencies, for example during the 2014–2016 Ebola outbreak in West Africa (5).

Mental health illnesses and drug abuse are common consequences observed during infectious disease outbreaks because of psychological distress, frustration, and unemployment (6, 49). There are concerns that the COVID-19 pandemic will lead to a mental health crisis, especially in countries with high disease burden (7). Unpredictability, uncertainty, disease severity, misinformation, and social isolation all contribute to stress and mental morbidity (8). Asmundson and Taylor (9, 10) and Xiao et al. (11) reported anxiety to be the most common of individual mental health symptoms, associated with impaired sleep. Mitsuishi and Ries (12) reported that stigmatization from an infectious disease such as COVID-19 could lead to depression in those afflicted.

Elevated levels of stress or anxiety as a function of disruption of livelihoods that lead to an increase in levels of depression, loneliness, harmful alcohol use, self-harm, or suicidal behavior which interferes with how people adjust in difficult situations in this era of the pandemic (1). Studies assessing the mental health status of respondents in developing countries are however, scarce. Common stressors include the risk of being infected and infecting others, health anxiety manifesting in form of mistaking common symptoms of other health problems such as fever for COVID-19, leading to fear of infection (13, 14).

Constant fear, worry and uncertainty combined with individual stressors within the population is likely to have long-term consequences within communities, families and vulnerable individuals. This manifests in the form of possible higher emotional state, anger and aggression against government, frontline workers, partners, and children (13, 14) and sometimes, fear and behaviors are modeled by ignorance, and misinformation (13, 14).

Low-income earners are defined as individuals in work whose income falls below the poverty line and an average Ugandan survives on < $1 US per day (15). Economic pressure associated with the pandemic has not been explored in low resource settings, thus undermining the mental health status of most Africans. This is important since persons in these communities work longer hours, earn less, and have a high dependent-income ratio (16). This study aimed to assess the mental health status among low income earners in Bushenyi district of southwestern Uganda during the first total COVID-19 lockdown.

## Methodology

### Study Site and Design

**Figure 1:**
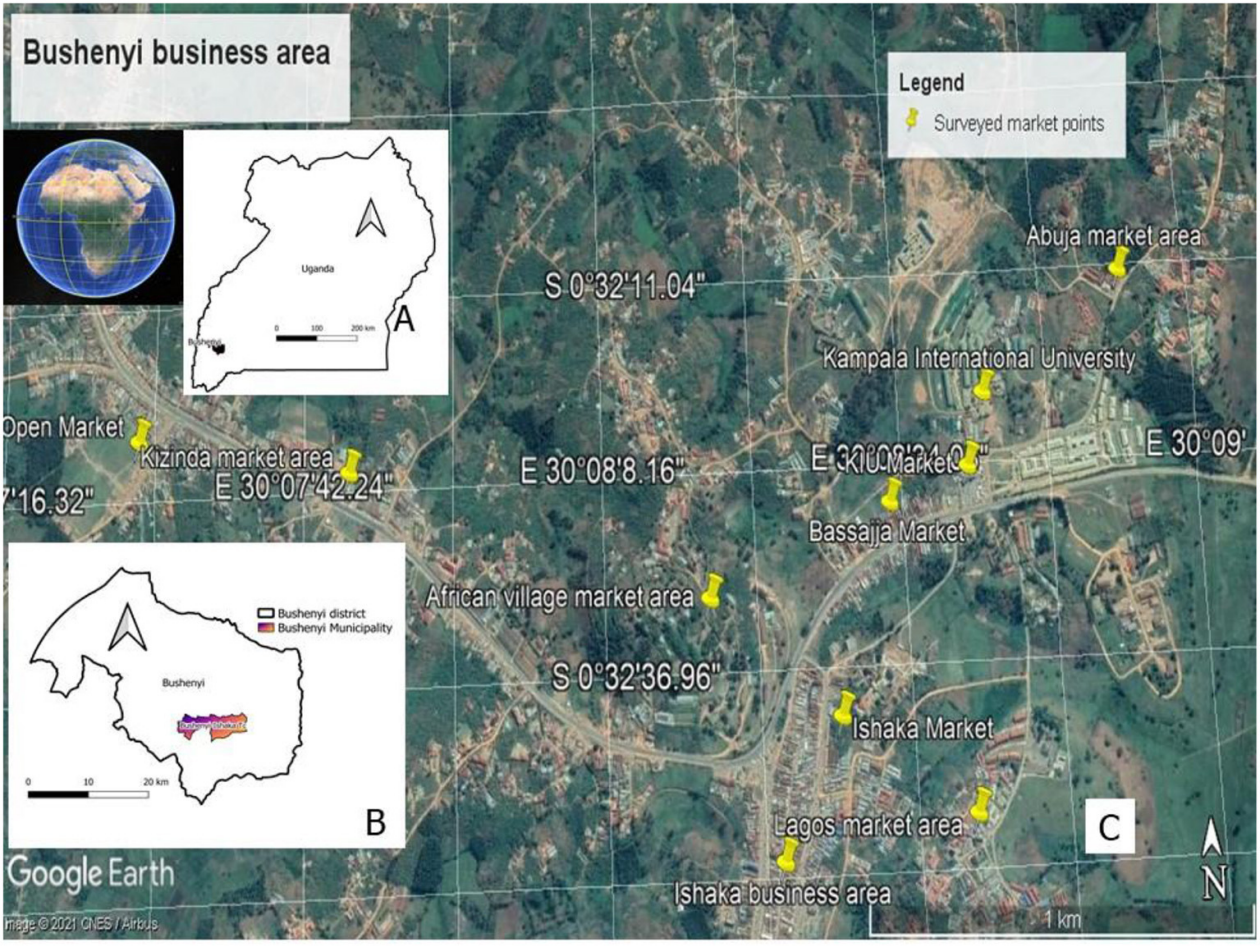
Description of the study area. The study was conducted in Uganda **(A)** which is an East African country. In particular, the surveyed participants where located in Bushenyi Municipality **(B)**, covering a total of 10 market areas **(C)**. Market areas visited included: Kizinda market area, Kizinda open market, African village market area, Ishaka business area, Ishaka open market, Bassajja market, Kampala International University (KIU), KIU open market, Lagos market area, and Abuja market areas **(C)**.

This was a cross-sectional study conducted amongst low-income earners i.e., boda-boda riders, taxi drivers, taxi turn-boys in the taxi-park, market sellers, barbers, hairdressers, photocopier attendants, and street food sellers in Bushenyi district (Figure 1).

### Inclusion and Exclusion Criteria

#### Inclusion Criteria

The study population consisted of males and females (*n* = 253) above 18 years of age, who have resided in the region for the past 12 months. This was important since these had permanent businesses and were residents in the area, thus able to assess how the pandemic has changed their work routine.

#### Exclusion Criteria

Individuals who refused to participate in the study or casual laborers and those not involved in the financial management of the business. In addition, individuals who owned other businesses outside the study area, although residents in the area were excluded from the survey.

### Data Collection and Management

#### Data Collection Method

An electronic questionnaire was shared with participants to assess their mental health, anger, anxiety, and depression status. Awareness of mental health care was assessed using simple questions and each of the options provided were assigned scores. Anxiety was assessed using a modified generalized anxiety disorder (GAD-7) item tool (17). Each of the responses attracted scores. Anger was assessed using a modified Spielberger's State-Trait Anger Expression Inventory-2 (STAXI-2) (18). Responses for each question were assigned scores. Depression was assessed using a modified Beck Depression Inventory (BDI) (19). Responses for each of the questions were assigned scores. The questionnaire consisted of multiple-choice unambiguous questions, and respondents who could not understand the questions had the questions interpreted in the local language for them by team members who could communicate effectively in the local vernacular. A Google format of the questionnaire was used to minimize physical contact and maintain social distancing according to the guidelines by WHO and the Ministry of Health in Uganda (Supplementary Material 1).

#### Data Management and Organization

Data from the survey was entered into Microsoft Excel (2016) and scores were assigned to each option as follows: Mental Health Care Awareness (Q5 – Q10): Numerical values – Mental Health Awareness [Correct response = 1, Incorrect response = 0]. Modified GAD Assessment of Anxiety (Q11 – Q16): Numerical values – Multiple response [For each option selected = 1, indifferent = 0].Modified STAXI-2 Assessment for Anger (Q17 – Q23): Numerical values – Multiple response [For each option selected = 1, indifferent = 0].Modified BDI Assessment for Depression (Q24 – Q30): Numerical values – Single graded response [Highest grade of 3, indifferent = 0].

Anxiety, Anger, and Depression were the mental health concerns of interest, while awareness was an influence variable of significant interest. These variables were obtained using interviewer-based questionnaires. The data were assessed for completeness and responses failing to meet the 75% cut-off (on all valid questions) were excluded. Scores of the multiple options for the modified GAD, and STAXI-2 were obtained by assigning one (1) mark per response, and the averages were obtained by summing all scores (qt) and dividing the number of questions (n). While BDI had four (4) options graded as 3, 2, 1, and 0 (for indifferent). For specific graded questions (yes, sometimes, or no), scores; 2, 1, 0 were assigned and all questions in this form were cumulated (per row), added, then divided by the maximum assigned weight (w) and the distribution (n). This provided the score (Level-score), which is then rated (name of condition; awareness, anxiety, depression, or anger) using cut-offs. To determine the level of awareness and mental state, the composite scores were grade; awareness (no; <0.3, low; 0.30–0.69, high; ≥70) and mental state (no/mild; <0.20, moderate; 20–0.49 or severe; ≥50). This scoring and grading system allows for both linear and uni/multivariate logistic regression analysis.

#### Statistical Analysis

Data was transferred to Minitab® 18.1 (Minitab, Inc. 2017, Pennsylvania, USA) and all relevant analysis was carried out. The data was not uniformly distributed and principal component analysis (PCA) was used to observe the uncorrelatedness of the dependent variables and determine how they account for differences and separated the socio-demographic variables into components. Spearman Rho correlation was used to observe the relationship between age, awareness, anxiety, anger, and depression, then all significant correlates were regressed using a system-assisted regression model. All analyses were performed at a 95% confidence level and *p*-values < 0.05 were taken to be significant.

## Results

### Sociodemographic Characteristics (Sex and Age-Group), Anxiety, Anger, and Depression

The study comprised of 150 males (59.3%) and 103 females (40.7%), with more middle-aged adults (170/253; 67.2%) compared to younger (65/253; 25.7%) and older (18/253; 7.1%) adults. The proportion of low-income earners in western Uganda with awareness of mental health care was 126/253 (49.8%). A high proportion of low-income earners reported to have experienced severe anxiety 174/253 (68.8%), moderate depression 144/253 (56.9) and moderate anger 153/253 (60.5) (Table 1).

**Table 1:** Response and factor information.

**Variables**	**Value/level**	**Frequency (%)**	**Median (95% CI)**
Sex	Male	150 (59.3)	
	Female	103 (40.7)	
Age (adults)	Young (18–24 years)	65 (25.7)	23 (22.0–23.9)
	Middle-aged (25–40 years)	170 (67.2)	30 (29.0–31.0)
	Older (>40 years)	18 (7.1)	47 (43.3–57.7)
Awareness	High	126 (49.8)	43.7–66.0
	Low	62 (24.5)	19.5–30.1
	No	65 (25.7)	20.6–31.3
Anxiety	Severe	174 (68.8)	62.9–74.3
	Moderate	74 (29.2)	23.9–35.1
	No/Mild	5 (2.0)	0.7–4.3
Anger	Severe	85 (33.9)	28.0–39.6
	Moderate	144 (56.9)	50.8–62.9
	No/Mild	24 (9.5)	6.3–13.6
Depression	Severe	12 (4.7)	2.6–7.9
	Moderate	153 (60.5)	54.4–66.4
	No/Mild	88 (34.8)	29.1–40.8
**Total**		**253**	

**Figure 2:**
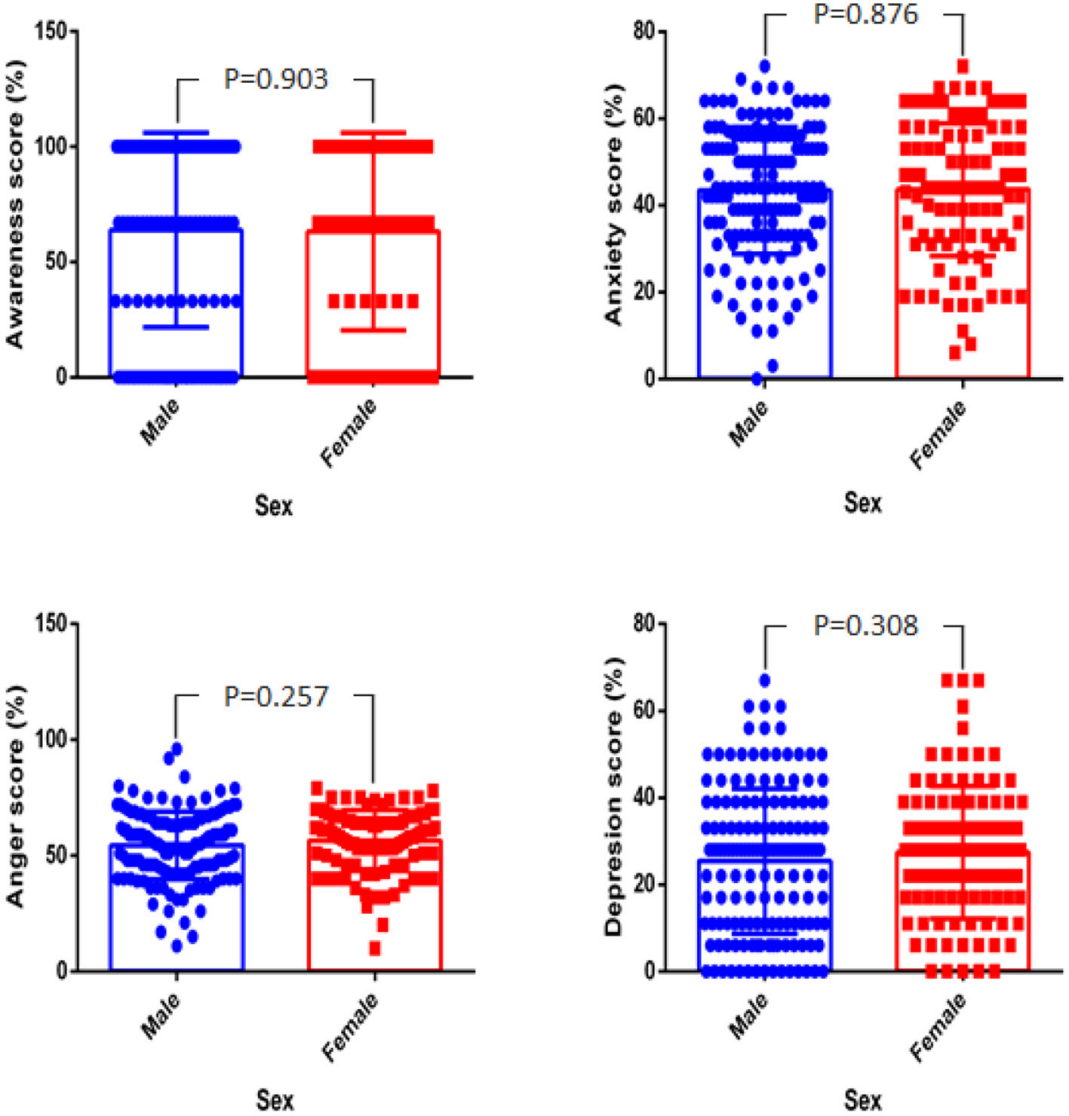
Percentage score difference for awareness, anxiety, anger, and depression between male and female.

The mean percentage score for awareness in male and female respondents were 63.8 and 63.2%, respectively. Female respondents reported anxiety, anger, and depression of 43.6, 56.5, and 27.5%, respectively, while male respondents reported anxiety, anger, and depression of 43.4, 54.5, and 25.4%, respectively (Figure 2).

**Figure 3:**
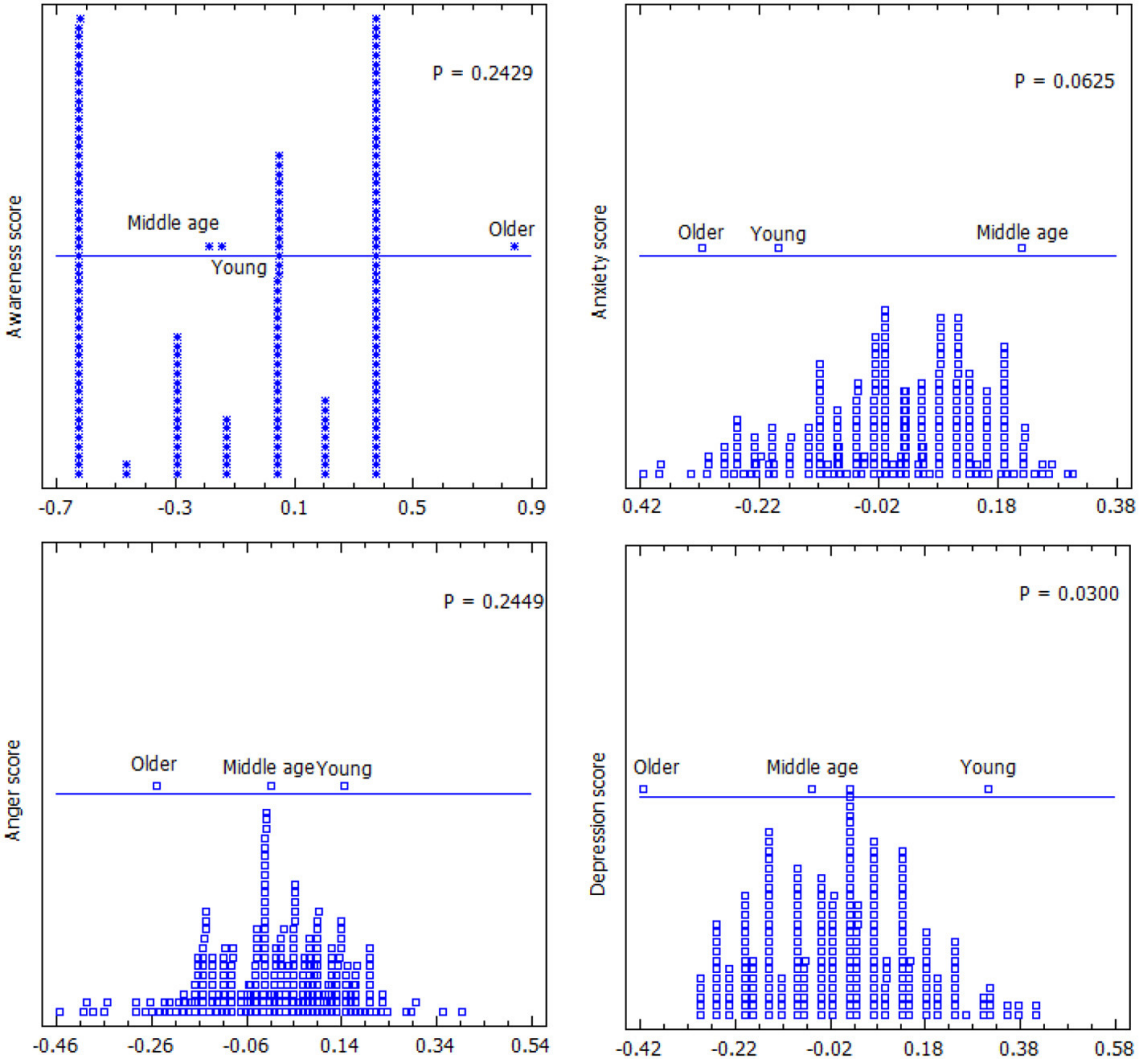
Composite score difference for awareness, anxiety, anger, and depression between the different age groups.

The study also showed that older adults had higher awareness of mental health, lower anxiety, anger, and depression than middle-aged and younger adults (Figure 3); however, the difference in the scores was only significant for depression (*P* = 0.03).

### Interactions Between COVID-19 Related Anxiety, Anger, and Depression Amongst Low-Income Earners in Uganda

The correlation analysis of the study variables was presented in Table 2. There was a negative relationship between age and depression (*r* = −0.154, *P* = 0.014), while a positive relationship was observed between anxiety and anger (*r* = 0.254, *P* < 0.001), anxiety and depression (*r* = 0.153, *P* = 0.015) and anger and depression (*r* = 0.153, *P* = 0.015).

**Table 2:** Spearman Rho correlation of age, awareness, anxiety, and anger on COVID-19 amongst low-income Ugandans.

**Correlations**	**Age**	**Awareness**	**Anxiety**	**Anger**
Awareness	0.049			
*P*-value	0.440			
Anxiety	0.081	−0.091		
*P*-value	0.199	0.151		
Anger	−0.024	0.033	0.245	
*P*-value	0.707	0.602	<0.001	
Depression	−0.154	−0.076	0.153	0.153
*P*-value	0.014	0.227	0.015	0.015

**Figure 4:**
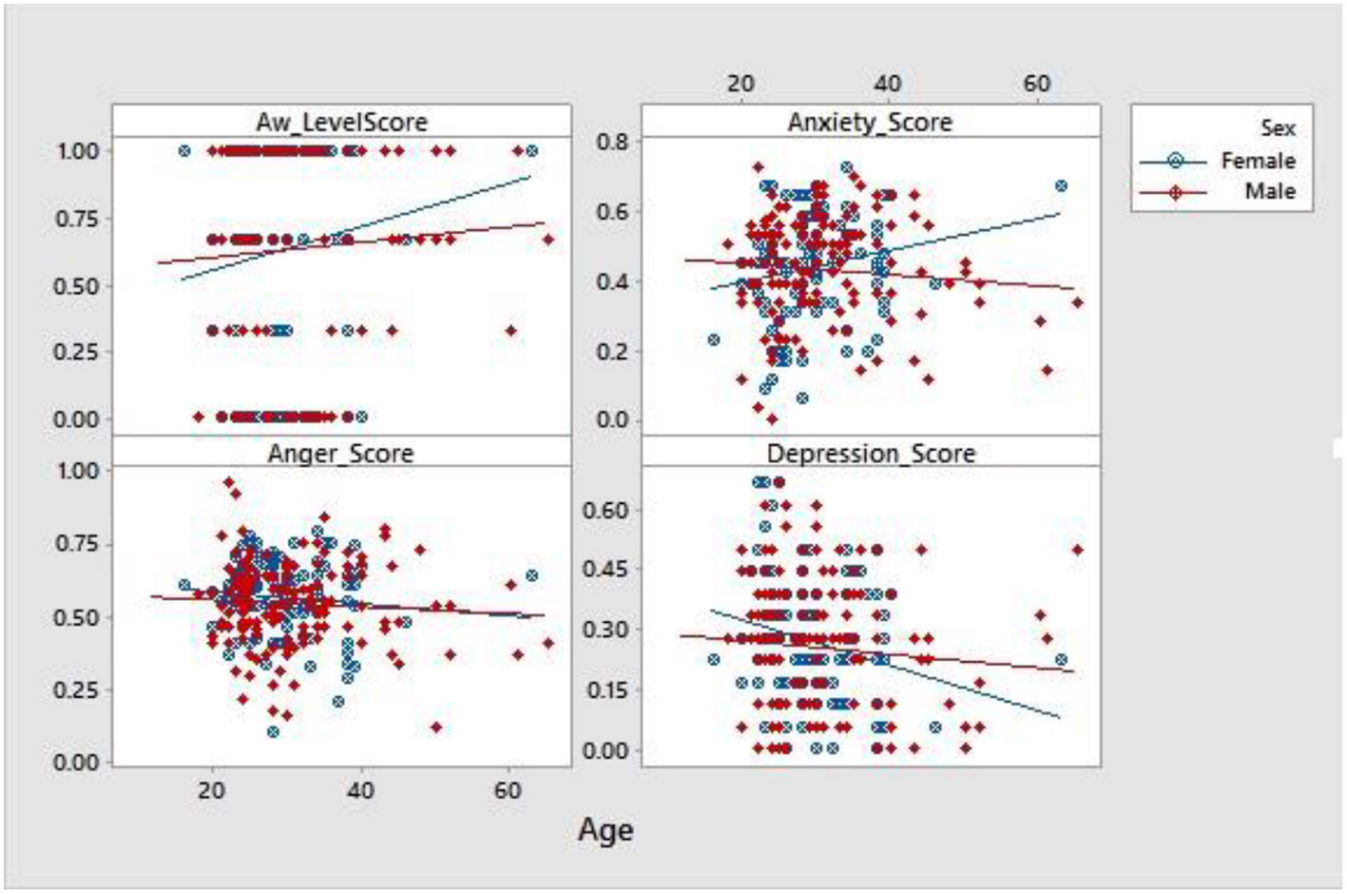
Scatter plot of Sex influence on age associated changes in awareness, anxiety, anger, and depression.

The study also showed that there were relationships in participants anger score however all other variables were not related in both sexes (Figure 4).

## Discussion

Survivors of natural disasters as exemplified by the current COVID-19 pandemic often suffer from traumatic experiences, anxiety disorders (GAD), depression, panic disorders and substance abuse (20–22). The study provided evidence that generally the Ugandan population was aware of mental health issues and this was in agreement with previous studies amongst healthcare workers (23) and market workers (24).

Females who demonstrated severe anxiety, moderate anger and depression probably due to their relatively low knowledge as compared to males on COVID-19. Social-economic barriers in several developing countries have continued to predispose women to apathy, segregation and mistreatment at the hands of their men counterparts in several rural communities in Africa, thus accounting for the severe discrepancies in knowledge between the two sexes. The WHO has identified women as vulnerable persons in Africa who should be supported in response programs against COVID-19 (25) and this is re-emphasized by the current study. Findings in the study were elderly persons showed a keen awareness and interest on mental healthcare demonstrated their keen interest to improve on their lifestyle, contrary to young adults in this population.

Anxiety also increased with age amongst females due to the inherent responsibilities associated with women in African communities. A majority of homes in Africa are managed by single mothers, and elderly women taking care of orphaned grandchildren, thus social pressures associated with COVID-19 were amplified in this population group than in their male counterparts who generally live more reckless lifestyles (i.e., alcoholism, polygamy, and crime, vices not common with African women).

Anger and depression decreased with an increase in age in the study population. These findings though perplexing, help to demonstrate the fluidity associated with community health response projects, and the need for flexibility while dealing with different age populations. Generally, Africa has a young vibrant population and these findings may not be applicable in the global context. Females were found to be more depressed probably due to the associated pressures of lost time due to the total lockdown which led to a closure of all academic centers of learning, forcing many young girls into early marriage and teenage pregnancies, thus derailing their academic goals (25). In addition, the study identifies Ugandan females as vulnerable persons to depression and in great need of guidance and counseling services for the promotion of better public health policies (26, 48). Generally, females experience more stress than males on a monthly basis as a result of physiological processes associated with their gender, thus offering a foundation for the increased anxiety attacks common in the gender (27, 28). Furthermore, neurophysiology studies have revealed that there are structural and functional differences in the brain relevant to anxiety in males and females (29–31) and blood pressure and pulse have been reported to be more reactive to anxiety in females compared to males (32), although these were not investigated in the current study.

A strong relationship between anxiety and anger was found amongst the young study participants probably due to frustrations associated with the lockdown and the fact that a majority of the youth in Uganda are under/unemployed. Our findings are in agreement with Walsh et al. (47) who reported a similar relationship between anxiety and anger in younger people. Poverty amongst the young and the on-going presidential elections were COVID-19 guidelines are enforced by the police against the opposition parties could have played a role in saturating a hateful atmosphere (personal communication). Amongst the youth, anger is a psychological and physical health predictor (33), demonstrating a need for policymakers to work closely with this age group.

Anger refers to a basic and universal emotional state brought forth by a perception of threat and it is associated with cognitions centered on others' transgressions (34). In contrast, irritability is a physiological state which is characterized by a lowered threshold for responding with negative affect to stimuli, often with anger and aggression (35, 44). Anger has not been included as a feature or symptom associated with anxiety disorders during the diagnostic process, however, irritability has been included as part of generalized anxiety disorders (GAD) diagnosis. Evidence has emerged in samples of adults with anxiety disorders that the rate and intensity of anger are elevated when compared to healthy individuals and this is common during severe anxiety (36). Anger may be expressed differently depending on anxiety diagnosis. For instance, it was observed that individuals with GAD may experience angry feelings but suppress them more than healthy controls, leading to higher levels of anxiety severity in adulthood (37, 46). This simply means that younger people express anger more than adults because they cannot suppress it, while adults tend to suppress anger and this increases anxiety. In addition, the initial increase in anger-score may be due to the inability or difficulty for younger persons to rate anger and anxiety as distinct constructs. This supports the study done by Walsh et al. (47) which reported that younger people confused physical symptoms of anxiety which mimic some physical symptoms of anger for anger and this led to difficulty in rating anger and anxiety as separate constructs.

A positive relationship was found between anxiety and depression in the study showing that younger persons (20 years of age) experienced depression as anxiety score increased more than adults (40–60 years of age) in the study. This supports the statistics from the Substance Abuse and Mental Health Services Administration (SAMHSA) of 2017 which estimated depressive disorder to be 7.1% for adults and 13.3% for adolescents/teens (45). Anxiety disorders and major depression occur during development with anxiety disorders beginning during pre-adolescence and early adolescence, while major depression tends to emerge during adolescence and early to mid-adulthood (38–40). Further studies have shown that anxiety disorders generally precede the presentation of major depressive disorder (39). Previous studies done in the USA revealed that depression is on the rise amongst teenagers and that females were three times more likely to experience depression compared to males. A 10-year study between 2007 and 2017 in the US revealed that the number of teenagers who experienced depression had increased by 59% and the rate was faster for females (66%) than males (44%) (41); the number of adults who experienced depression increased by 7% within the same period. Academic and social pressures were cited by experts as the reasons behind the increase in depression among the teens recruited in that study. About six out of 10 (61%) of the teens reported they felt a lot of pressure to get good grades while 29% of the teens reported they felt a lot of pressure to look good and 28% of the teens reported they felt pressured to fit socially (41). The results from our study showed that people at 20 years of age (this age bracket captures teens), had a depression score which increased as anxiety score increased. In Bushenyi district (Uganda), some of the students within the 20 years of age group do engage in low-income jobs after school hours and this may affect their academic performances or social life resulting in depression if not monitored and this observation is in agreement with postulations by Geiger and Leslie (41).

There was a positive relationship between anger score and depression score in our study. It was observed that as anger score increased depression score tends to increase. Anger is one of the ways that depression manifests and there is an association between the level of anger that people experience and the severity of depression (50). According to the Anxiety and Depression Association of America (ADAA), depression is expressed in different ways by different people. Females with depression tend to feel sad or guilty while males with depression are more likely to feel irritable and angry (ADAA). However, there is limited research available to support or show that anger can cause depression.

A general observation from our study showed that awareness of mental health care was high (49.8%) across the sample population (*n* = 253) and an increase in age-related to high awareness. 24.5% of the respondents had low awareness and 25.7% had no awareness of mental health care. 68.8% of the respondents experienced severe anxiety due to the COVID-19 lockdown and 29.2% experienced moderate anxiety while 2.0% experience no/mild anxiety based on anxiety score scale using a modified GAD-7 item tool. Also, 33.9% of the respondents experienced severe anger due to the COVID-19 lockdown, and 56.9% experienced moderate anger while 9.5% experienced no/mild anger based on the anger-score grading scale using a modified STAXI-2 item tool. About 4.7% of the respondents experienced severe depression due to the COVID-19 lockdown and 60.5% experienced moderate depression while 34.8% experienced no depression based on the depression score grading scale using a modified BDI. Other studies done elsewhere have reported different figures due to the variations in the study population and sample size. Wang et al. (42), in their study of mental health status related to COVID-19 among the general population (*n* = 1,210) in China reported that 16.5% experienced moderate to severe depression symptoms, 28.8% experienced moderate to severe anxiety symptoms and 8.1% experienced moderate to severe stress. The variation in statistics from our study in Uganda and Wang et al. (42) in China maybe because we targeted low-income earners which are persons who depend on day-to-day-business to make ends meet, tools used for assessment, and sample size. However, our study indicates that anxiety was the most common mental health challenge and this supports the study by Wang et al. (42), which also identified anxiety as the most common mental health challenge in their study. Other studies have reported increased traumatization related to COVID-19 among the public and non-front-line nurses in China (43). Xiao et al. (11), targeted individuals (*n* = 170) observing COVID-19 self-isolation for 14 days in China, and they reported that anxiety positively correlated with stress and negatively with sleep quality and social capital and social capital positively correlated with sleep quality. This indicates that COVID-19 lockdown, without doubt, affects the mental health status of individuals.

## Conclusion

The study showed that pandemic mental health is a realistic public health concern which needs to be addressed. Poor and low knowledge on the pandemic precipitates anxiety which leads to a depressed population. In particular, females and the elderly were identified as vulnerable persons who should be prioritized in any community extension activities. The authors could not include the COVID-19 status of the respondents due to absence of clinical data at the time of the study (early COVID-19 period and no community testing was in place). We also did not have access to population data to access clinical mental status of study participants prior to the pandemic. Further studies to generate information on the impact of the pandemic in the general population could help guide policymakers draft community-practical policies which address mental health amongst the vulnerable communities of Uganda.

## Data Availability Statement

Data files can be accessed at https://figshare.com/s/88a0cf974d67f2654816.

## Ethics Statement

The studies involving human participants were reviewed and approved by Kampala International Ethical Review Board Kampala International University, Uganda. The patients/participants provided their written informed consent to participate in this study.

## Author Contributions

VA and IU conceptualized the study. IU, KK, FS, and SW designed the study. VA, IU, RS, AM, ETA, VN, JTA, and FS collected the data. VA, IU, and EOA conducted statistical analysis. VA, IU, KK, EOA, IJ, SH, EM, HN, and SW conducted data interpretation. VA, IU, and KK drafted the initial manuscript while EOA, IJ, AM, RS, GC, ST, OO, NM, AA, JOA, AMA, SO, FS, ETA, JTA, VN, HY, CO, SH, EM, HN, and SW reviewed it for intellectual content. All authors approved the final version for publication and remain in agreement to ensure that questions related to the integrity of any part of the work are resolved.

## Funding

This work was supported by Zhejiang University Education Foundation Emergency Research Fund (SW and KK); Global Challenges Research Fund and the University of Edinburgh.

## Conflict of Interest

The authors declare that the research was conducted in the absence of any commercial or financial relationships that could be construed as a potential conflict of interest.

## Publisher's Note

All claims expressed in this article are solely those of the authors and do not necessarily represent those of their affiliated organizations, or those of the publisher, the editors and the reviewers. Any product that may be evaluated in this article, or claim that may be made by its manufacturer, is not guaranteed or endorsed by the publisher.

## Supplementary Material

Supplementary Material 1 The questionnaire.
