# Supplementary material for: Anxiety, Anger and Depression Amongst Low-Income Earners in Southwestern Uganda During the COVID-19 Total Lockdown
Source: Front Public Health. 2021 Dec 9;9:590458. doi: 10.3389/fpubh.2021.590458 (PMC8695878; doi:10.3389/fpubh.2021.590458)
Supplement: Supplementary Material 1 — The questionnaire. [file Table_1.DOCX]

**Questionnaire: Mental health effect of Covid 19 lockdown**

1. Sex

- Male{ }
- Female{ }

2. Age__________

3. Educational status

- Primary level{ }
- Secondary level{ }
- Tertiary level{ }
- No formal education{ }

4. Marital status

- Single{ }
- Married{ }
- Separated{ }
- widowed{ }

**ASSESSMENT OF MENTAL HEALTH CARE AWARENESS**

5. Do you know what mental health care is?

- Yes{ }
- No{ }

6. Do you know any facility in Uganda where mental health care is provided?

- Yes{ }
- No{ }

7. Is there a place in your locality where mental health care is provided?

- Yes{ }
- No{ }

8. Do you have a pre-existing mental health challenge?

- Yes{ }
- No{ }
- Not sure{ }

9. Which of the following mental health challenges applies to you?

- Anxiety{ }
- Depression{ }
- Paranoia{ }
- Anger issues{ }
- Others{ }
- Not available{ }

10. How do you handle your mental health challenge? (tick one or more)

- Use of medication{ }
- Drinking alcohol or smoking{ }
- Watching movies{ }
- Singing or dancing{ }
- Binge eating{ }
- others{ }
- None of the above{ }

**ASSESSMENT OF ANXIETY USING MODIFIED GAD**

11. Have you heard of any mental health challenge related to Covid-19 before?

- Yes{ }
- No{ }
- Maybe{ }

12. How has the Covid-19 updates and stories globally affect you? (tick one or more)

- Makes me feel nervous or anxious{ }
- Makes me worried{ }
- Makes me restless or sleepless{ }
- Makes me easily annoyed or irritable when some discusses it{ }
- Makes me afraid I may become infected{ }
- Indifferent { }

13. Since Covid-19 was confirmed in Uganda, how much have you adhered to Ministry of Health specified safety

- obsessively{ }
- moderately{ }
- slightly{ }
- not sure{ }

14. How do you feel if you unconsciously fail to observe or adhere to the ministry of health specified safety guidelines? (tick one or more)

- Makes me feel nervous or anxious{ }
- Makes me worried{ }
- Makes me restless or sleepless{ }
- Makes me easily annoyed or irritable when some discusses it{ }
- Makes me afraid I may become infected{ }
- Indifferent { }

15. Has the lockdown affected your source of income?

- Yes{ }
- No{ }
- Prefer not to say{ }

16. If the lockdown has affected your source of income, how does that make you feel? (Tick one or more)

- Makes me feel nervous or anxious{ }
- Makes me worried{ }
- Makes me restless or sleepless{ }
- Makes me afraid that I may lose my job
- Indifferent { }

**ASSESSMENT OF ANGER USING MODIFIED STAXI-2**

17. How does the lockdown make you feel? (tick one or more)

- I feel Angry{ }
- I feel furious{ }
- I feel like hitting or kicking something
- I feel irritated{ }
- I feel annoyed{ }
- I feel mad{ }
- I feel like breaking things{ }
- Indifferent { }

18. If you are a parent with young children, how does this lockdown with little ones make you feel? (tick one or more)

- I feel Angry{ }
- I feel furious{ }
- I feel like hitting or kicking something
- I feel irritated{ }
- I feel annoyed{ }
- I feel mad{ }
- I feel like breaking things{ }
- Not applicable{ }

19. Has the lockdown made the young children staying with you to spoil or break any household items and how does that make you feel? (tick one or more)

- I feel Angry{ }
- I feel furious{ }
- I feel like hitting or kicking something
- I feel irritated{ }
- I feel annoyed{ }
- I feel mad{ }
- I feel like breaking things{ }
- Not applicable{ }

20. Have you been spending more on feeding during the lockdown?

- Yes{ }
- No{ }
- Not sure{ }

21. If yes, how does that make you feel? (tick one or more)

- I feel Angry{ }
- I feel furious{ }
- I feel like hitting or kicking something
- I feel irritated{ }
- I feel annoyed{ }
- I feel mad{ }
- I feel like breaking things{ }

22. Are you worried about your finances or foodstuffs finishing during the lockdown?

- Yes{ }
- No{ }
- Not sure{ }

23. If yes, how does that make you feel? (tick one or more)

- I feel Angry{ }
- I feel furious{ }
- I feel like hitting or kicking something
- I feel irritated{ }
- I feel annoyed{ }
- I feel mad{ }
- I feel like breaking things{ }
- Indifferent { }

**ASSESSMENT OF DEPRESSION USING MODIFIED BDI**

24. How do you feel about your sleeping pattern since the lockdown began?

- I do not feel sad about it{ }
- I feel sad about it{ }
- I am sad all the time and can’t snap out of it{ }
- I am so sad and unhappy about it
- Indifferent { }

25. Has this lockdown affected your weight?

- I haven’t lost much weight if any{ }
- I have lost more than 2.5kg{ }
- I have lost more than 5kg{ }
- I have lost more than 7.5kg{ }
- Not sure{ }

26. If you have gained more weight, how does that make you feel?

- I do not feel sad{ }
- I feel Sad{ }
- I feel unhappy{ }
- I feel annoyed{ }
- Indifferent { }

27. How has the lockdown affected your zeal towards socializing?

- I have not lost interest in socializing with people{ }
- I am less interested in socializing with people than I used to be{ }
- I have lost most of my interest in socializing with other people{ }
- I have lost all my interest in socializing with other people{ }
- Did not affect if{ }

28. What has been your coping mechanism to maintain your mental health during the lockdown? (Tick one or more)

- Watching movies{ }
- Reading { }
- Engaging in meditation{ }
- Personal development in form of learning new skills{ }
- Eating more than I used to{ }
- Others{ }

29. How satisfied are you with what you earn monthly from your work or business?

- I do not feel like a failure{ }
- I feel like I have failed myself{ }
- I wished I could get a better job or pay{ }
- I feel I am a complete failure as a person
- Indifferent { }

30. If covid-19 doesn’t have a cure or vaccine, how do you feel about the future?

- I am not particularly discouraged about the future{ }
- I feel discouraged about the future{ }
- I feel I have nothing to look forward to
- I feel the future is hopeless and that things cannot change { }
